# Correlational study on the levels of 25-hydroxyvitamin D and non-alcoholic fatty liver disease in type 2 diabetic patients

**DOI:** 10.1186/s12902-021-00762-1

**Published:** 2021-05-18

**Authors:** Yuanyuan Zhang, Juyi Li, Yingqun Ni, Yan Wang, Huaizhen Liu

**Affiliations:** 1 Department of Endocrinology, Geriatrics Center, The First Affiliated Hospital of Anhui University of Traditional Chinese Medicine, Hefei, 230001 Anhui China; 2 Department of Endocrinology, The First Affiliated Hospital of Anhui University of Traditional Chinese Medicine, Hefei, 230001 Anhui China

**Keywords:** Type 2 diabetes mellitus;non-alcoholic fatty liver disease;progressive liver fibrosis;25-hydroxyvitamin D

## Abstract

**Background:**

It is widely acknowledged that nonalcoholic fatty liver disease (NAFLD) and type 2 diabetes mellitus(T2DM) are all chronic metabolic diseases. The objective of this study is to retrospectively probe the association between the 25-hydroxyvitamin D (25-(OH)D) and NAFLD in type 2 diabetic patients.

**Methods:**

Three hundred thirty-nine T2DM patients participated in this research and from November 2018 to September 2019 and were divided into simple T2DM group (108 cases) and T2DM with NAFLD group (231 cases) in conformity with abdominal ultrasound diagnosis. The NAFLD fibrosis score (NFS) ≥0.676 was defined as progressive liver fibrosis.231 T2DM with NAFLD patients were categorized into two subgroups: progressive liver fibrosis subgroup (48 cases) and without progressive liver fibrosis subgroup (183 cases).

**Results:**

The prevalence of NAFLD by Abdominal ultrasonography was 68%.The results indicated that the levels of 25-(OH) D were significantly lower in T2DM with NAFLD group than that in simple T2DM group(*P* < 0.01). The levels of 25-(OH) D were significantly lower in progressive liver fibrosis subgroup than that in patients without progressive liver fibrosis and simple T2DM,and 25-(OH) D levels were lower in without progressive liver fibrosis subgroup than that in simple T2DM group(*p* < 0.01 or *p* < 0.05). Multivariate logistic regression analysis showed that levels of 25-(OH) D were negative correlation with risk of NAFLD and progressive liver fibrosis(*p* = 0.011、*p* = 0.044,respectively).

**Conclusions:**

we could come to a conclusion that low levels of 25-(OH) D was a risk factor for NAFLD and progressive liver fibrosis in T2DM patients.

## Background

Non-alcoholic fatty liver disease (NAFLD) is a kind of liver disease, which is characterized by abnormal accumulation of liver fat and insulin resistance (IR).

Clinically, the disease progress can be divided into five stages: nonalcoholic steatosis (NAS), non-alcoholic steatohepatitis (NASH), liver fibrosis, liver cirrhosis and hepatocellular carcinoma [1, 2]. The incidence of NAFLD and liver fibrosis was closely contacted to the increase of mortality of liver-related diseases [3]. The prevalence rate of NAFLD in the world was as high as 25.2% [4], while it increased by 27.4% in Asia [5]. The incidence of NAFLD that was screened by ultrasound in T2DM patients was 73.7% [6]. Diabetic patients with NAFLD were prone to NASH and liver fibrosis, and increased the mortality of liver-related diseases and the risk of cardiovascular diseases [7]. Therefore, we should pay attention to the progress of liver disease in T2DM with NAFLD patients. Many Scholars [8, 9] had proposed to use NAFLD scoring system (NFS) for evaluating the existence of liver fibrosis in NAFLD patients. This scoring system is non-invasive, which makes up for the defect of using invasive liver biopsy in the past.

Vitamin D is a kind of steroid hormone [10], which plays an important role in anti-inflammation, anti-oxidation, anti-fibrosis, immune regulation and so on, and.

participates in the occurrence and development of chronic liver diseases [11]. Ding et al. [12] conducted basic research on vitamin D receptor knockout mice and found that it could spontaneously produce liver injury and liver fibrosis. Clinical studies [13] also found that the low levels of 25-(OH) D were related to the occurrence of progressive liver fibrosis. However, some scholars have concluded that the low levels of 25-(OH) D had nothing to do with NAFLD [14] and liver fibrosis [15]. Vitamin D also plays an important role in altering the risk of T2DM, such as mediating β -cell function, insulin sensitivity, and systemic inflammation [16]. On the basis of the controversy over the relationship between vitamin D and NAFLD,and few studies are conducted on that population of T2DM combined with NAFLD, so we aimed to explore the relationship between 25-(OH) D and NAFLD by observing serum 25-(OH) D levels in patients with T2DM in this study.

## Methods

### Research subjects

This study is a retrospective research of 580 T2DM patients admitted to the endocrinology department of our hospital,from November 2018 to September 2019.The detection of T2DM made full use of the diagnostic criteria proposed by the WHO Diabetes Expert Committee in 1999.Exclusion criteria: taking drugs affecting vitamin D levels(*n* = 14), excessive drinking(*n* = 56), malignant tumor(*n* = 9), pregnancy.

(*n* = 4) and type 1 diabetes(*n* = 5), acute complications of diabetes(*n* = 25), acute cardiovascular and cerebrovascular events(*n* = 35), serious liver and kidney dysfunction(*n* = 26), thyroid diseases(*n* = 32), viral liver diseases(*n* = 6), alcoholic liver diseases(*n* = 20), autoimmune liver diseases(*n* = 2), drug-induced liver diseases(*n* = 4) and other acute and chronic liver diseases(*n* = 3). Finally, 339 T2DM patients (192 men and 147 women) were included in the study.

### General clinical data and laboratory examination indicators

We collected general information of gender, age, height, weight, diabetic duration, drinking history, past medical history (tumor, liver disease, thyroid disease, etc.),waist circumference, systolic pressure (SBP) and diastolic pressure (DBP). All patients were collected samples of venous blood on an empty stomach. Using tubes coated with coagulant to contain whole blood and centrifuge it. After separation of serum, alanine aminotransferase (ALT), aspartic acid aminotransferase (AST) and glutamyltransferase (GGT) were detected by rate method;The HMMPS method for the determination of creatinine (CR);Uric acid (UA) was detected by uricase method;Bromocresol green method for the determination ofalbumin (ALB); Fasting blood glucose (FBG) was measured by hexokinase method;Triglycerides were detected by GPO enzyme reagent method;Enzymatic oxidation of cholesterol for the determination of total cholesterol (TC);Antibody blocking method for the determination of high density lipoprotein (HDL);Choice protection method for the determination of low density lipoprotein (LDL);Transmissivity turbidimetry method for the determination of cystatin C (CysC);Fasting C-peptide (FCP) was detected using magnetic particle chemiluminescence method. The specificity of this method is not higher than 0.2 ng/ml, and the coefficient of variation is not higher than 15%.Using high performance liquid chromatography-tandem mass spectrometry was to measure 25-(OH)D.In addition, tubes coated with EDTA-2 anticoagulant were used to contain blood samples. High-performance liquid chromatography for the determination of glycosylated hemoglobin (HbA1c); flow cytometry (Sysmex XN9000) for the determination of platelet (PLT).

### Definition, calculation and grouping

NAFLD was detected as abdominal color ultrasound [17] which was operated by senior physician in our hospital. The imaging diagnosis of fatty liver needs to satisfy the following ultrasound findings: high echo in the proximal diffusing point of the liver, higher echo intensity in the liver than in the kidney, unclear intrahepatic tube structure. Under this situation, the distant echo of liver intend to become weaker and weaker. The diagnosis of NAFLD must meet the following conditions:no history of drinking.;excluding some key acute and chronic liver diseases, unexplainable continuous increase of serum indices of liver function for more than 6 months [18].

The body mass index (BMI) was calculated by using the body weight (kg) and the square of the height (m^2^). The value of modified homeostasis model assessment for insulin resistance(C-peptide)(HOMA-IR (CP)) was calculated by FCP instead of fasting insulin, HOMA-IR (CP) = 1.5 + FBG (mmol / L) x FCP (pmol / L) / 2800 [19]. NFS was defined as NFS = (− 1.675 + 0.037 × age (year) + 0.094 × BMI (kg/m2) + 1.13 × impaired fasting glucose/presence of diabetes (yes = 1, no = 0) + 0.99 × AST/ALT ratio - 0.013 × platelet count (× 109/L) - 6.6 × albumin (g/dL) [9].

According to abdominal color ultrasonography, all patients were divided into two different groups, the first one was simple T2DM group(*n* = 108 cases) and the second one was T2DM with NAFLD group(*n* = 231 cases). According to the definition of NFS ≥ 0.676 as progressive liver fibrosis [9], 231 patients with NAFLD were divided into two subgroups: the first part patients were these without progression liver fibrosis subgroup(*n* = 183 cases) and the second part patients were these with progressive liver fibrosis subgroup (*n* = 48 cases).

### Statistical analysis

SPSS21.0 statistical software was used for the data analysis and the Kologorov-Smirnov normality of all data were tested. The measured data of the normal distribution was represented by mean ± SD.Comparisons were conducted between two groups and the comparing process was finished by making full use of independent T test,while the variance analysis was used for comparison among multiple groups. Measurement data for non-normal distributions were expressed as medians (interquartile intervals). Under this situation, two groups were compared by using the Mann-Whitney rank sum test,while Kruskal-Wallis test wass used for comparison among multiple groups, at the same time, LSD test was used for intra-group comparison. Counting data was shown by the number of cases, the Chi-square test was adopted to demonstrate the differences within two or more groups. The links between 25-(OH) D and NAFLD were analyzed by logitic regression. *P* < 0.05 or *P* < 0.01 represented the obvious differences in statistics.

## Results

### Comparison of general data and biochemical indexes in each group

The diagnosis of NAFLD by abdominal ultrasonography was 231 cases (68%) (Table 1). Patients in T2DM with NAFLD group were younger, shorter diabetic duration (all *p* < 0.05). Values of BMI,waist circumference, ALT, AST, GGT, fasting blood glucose (FBG), triglyceride (TG), triglyceride (TC), HOMA-IR (CP) and HBA1C values were all higher in T2DM with NAFLD group,while creatinine (CR), CysC, high density lipoprotein (HDL) and 25-(OH) D levels were significantly lower among the T2DM combined-NAFLD group than simple T2DM group (all *p* < 0.05).

**Table 1:** Comparison of general material and biochemical indexes of each group

	T2DM with NAFLD (*n* = 231)	Simple T2DM (*n* = 108)	*P* value
Sex (male/female)	134/97	58/50	0.456
Age (years)	57.2 ± 12.2	63.2 ± 10.8	0.000
Diabetic duration (years)	8.0 (3.0–13.0)	10.0 (5.0–15.0)	0.049
BMI (kg/m^2^)	26.88 ± 3.28	24.69 ± 3.38	0.000
Systolic pressure(mmHg)	132 (122–145)	132 (120–152)	0.533
Diastolic pressure(mmHg)	82.8 ± 10.2	82.1 ± 10.2	0.567
Waistline(cm)	93 (88–100)	90 (85–95)	0.000
ALT(U/L)	20 (15–32)	15 (12–22)	0.000
AST(U/L)	18 (15–24)	17 (14–21)	0.019
GGT (U/L)	30.0 (21.0–44.0)	18.5 (13.0–28.0)	0.000
CR (umol/L)	57.9 (49.2–68.8)	62.8 (49.8–72.3)	0.042
UA (umol/L)	324.1 ± 91.4	304.1 ± 101.6	0.072
CysC (mg/L)	0.91 (0.80–1.05)	0.97 (0.83–1.17)	0.018
FBG (mmol/L)	7.89 (6.15–11.01)	6.86 (5.59–9.84)	0.003
TG (mmol/L)	1.90 (1.25–2.73)	1.20 (0.89–1.58)	0.000
TC (mmol/L)	4.86 ± 1.10	4.56 ± 1.21	0.023
HDL (mmol/L)	1.01 (0.86–1.20)	1.10 (0.98–1.39)	0.000
LDL (mmol/L)	2.88 ± 0.83	2.69 ± 1.04	0.072
HOMA-IR (CP)	3.90 (3.10–4.97)	2.82 (2.45–3.67)	0.000
HBA1C (%)	8.5 (7.1–9.8)	7.7 (6.8–9.5)	0.023
25-(OH) D (ng/mL)	14.80 (11.52–18.37)	16.21 (12.22–22.59)	0.009

The measured data of the normal distribution was represented by mean ± SD. Measurement data for non-normal distributions were expressed as medians (interquartile intervals)

Note: *NAFLD* Nonalcoholic fatty liver disease, *T2DM* Type 2 diabetes mellitus, *BMI* Body mass index, *NFS* NAFLD fibrosis score, *ALT* Alanine aminotransferase, *AST* Aspartic acid aminotransferase, *GGT* Glutamyltransferase, *CR* Creatinine, *UA* Uric acid, *CysC* Cystatin c, *FBG* Fasting blood glucose, *TG* Triglyceride, *TC* Total cholesterol, *HDL* High density lipoprotein, *LDL* Low density lipoprotein, *Homa-IR (CP)* Homeostasis model assessment for insulin resistance(C-peptide), *HbA1c* Glycosylatedhemoglobin, *CR* Creatinine, *UA* Uric acid, *25-(OH) D* 25- hydroxyvitamin D

### Comparison of general data and biochemical indexes of each subgroup

According to the definition of NFS ≥ 0.676 as progressive liver fibrosis, 231 patients with NAFLD were divided into two subgroups: 183 cases without progressive liver fibrosis and 48 cases with progressive liver fibrosis,and simple T2DM patients form three groups (Table 2). There are significant differences in age, diabetic duration, BMI, waist circumference, ALT, AST, GGT, CR, UA, CysC, FBG, TG, TC, HDL, HOMA-IR (CP), 25-(OH) D and HBA1C among the three groups (*p* < 0.05 or *p* < 0.01). At the same time,age, AST, GGT, UA and HOMA-IR (CP) were all higher,while HDL and 25-(OH) D were lower in patients with progressive liver fibrosis subgroup that in simple T2DM group. Age,course of diabetes,CR, CysC, HDL and 25-(OH) D were lower,whlie BMI,waist circumference, ALT, GGT, FBG, TG, TC and HOMA-IR (CP) were higher in patients with non-progressive liver fibrosis subgroup that in simple T2DM group(*p* < 0.05 or *p* < 0.01). By comparing with patients in the non-progressive liver fibrosis subgroup,patients with advanced liver fibrosis were older, with longer course of diabetes,and the fact that the levels of AST, CR, UA and CysC were very high, but the amount of ALT and 25-(OH) D were much lower in patients in the advanced liver fibrosis subgroup (*p* < 0.05 or *p* < 0.01) .

**Table 2:** Comparison of general material and biochemical indexes of each subgroup

	T2DM with NAFLD *(n* = 231)		Simple T2DM (*n* = 108)	*P* value
	NFS < 0.676(*n* = 183)	NFS ≥ 0.676(*n* = 48)		
Sex (male/female)	107/76	27/21	58/50	0.729
Age (years)	54.4 ± 10.9^##^	67.9 ± 11.2**^△^	63.2 ± 10.8	0.000
Diabetic duration (years)	7.0 (3.0–12.0)^##^	11.0 (7.0–17.0)**	10.0 (5.0–15.0)	0.000
BMI (kg/m2)	26.60 ± 3.27^##^	27.93 ± 3.14*^△△^	24.69 ± 3.38	0.000
Systolic pressure(mmHg)	132 (122–144)	133 (123–155)	132 (120–152)	0.330
Diastolic pressure(mmHg)	83.2 ± 9.6	81.1 ± 12.0	82.1 ± 10.2	0.374
Waistline(cm)	93 (87–99)^##^	96 (90–101)^△△^	90 (85–95)	0.000
ALT(U/L)	21 (16–32)^##^	18 (13–30)*	15 (12–22)	0.000
AST(U/L)	18 (15–22)	20 (16–27)*^△△^	17 (14–21)	0.006
GGT (U/L)	31.0 (22.0–49.0)^##^	25.5 (18.0–42.8)^△△^	18.5 (13.0–28.0)	0.000
CR (umol/L)	56.0 (47.6–65.1)^##^	64.2 (53.7–79.9)**	62.8 (49.8–72.3)	0.000
UA (umol/L)	315.5 ± 91.9	356.9 ± 82.7**^△△^	304.1 ± 101.6	0.005
CysC (mg/L)	0.88 (0.79–0.98)^##^	1.09 (0.93–1.37)**	0.97 (0.83–1.17)	0.000
FBG (mmol/L)	8.21 (6.55–11.35)^##^	7.59 (5.70–9.89)	6.86 (5.59–9.84)	0.003
TG (mmol/L)	1.98 (1.31–2.84)^##^	1.68 (1.12–2.64)	1.20 (0.89–1.58)	0.000
TC (mmol/L)	4.90 ± 1.12^#^	4.69 ± 1.02	4.56 ± 1.21	0.037
HDL (mmol/L)	1.01 (0.86–1.17)^##^	1.05 (0.85–1.30)^△^	1.10 (0.98–1.39)	0.000
LDL (mmol/L)	2.90 ± 0.83	2.81 ± 0.85	2.69 ± 1.04	0.167
HOMA-IR (CP)	3.90 (3.10–5.13)^##^	3.67 (3.06–4.54)^△^	2.82 (2.45–3.67)	0.000
HBA1C (%)	8.7 (7.2–9.9)	8.2 (6.9–9.5)	7.7 (6.6–9.5)	0.042
25-(OH) D (ng/mL)	14.92 (11.72–18.64)^#^	13.97 (11.21–15.86)*^△△^	16.21 (12.22–22.59)	0.005

The measured data of the normal distribution was represented by mean ± SD. Measurement data for non-normal distributions were expressed as medians (interquartile intervals).*p < 0.05, **p < 0.01, T2DM with NFS ≥ 0.676 vs T2DM with NFS < 0.676; ^△^*p* < 0.05, ^△△^*p* < 0.01,T2DM with NFS ≥ 0.676 vs Simple T2DM;^#^*p* < 0.05, ^##^*p* < 0.01, T2DM with NFS < 0.676 vs Simple T2DM

Note: *NAFLD* Nonalcoholic fatty liver disease, *T2DM* Type 2 diabetes mellitus, *BMI* Body mass index, *NFS* NAFLD fibrosis score, *ALT* Alanine aminotransferase, *AST* Aspartic acid aminotransferase, *GGT* Glutamyltransferase, *CR* Creatinine, *UA* Uric acid, *CysC* Cystatin c, *FBG* Fasting blood glucose, *TG* Triglyceride, *TC* Total cholesterol, *HDL* High density lipoprotein, *LDL* Low density lipoprotein, *Homa-IR (CP)* Homeostasis model assessment for insulin resistance(C-peptide), *HbA1c* Glycosylatedhemoglobin, *25-(OH) D* 25- hydroxyvitamin D

### Analysis of influencing factors of NAFLD and progressive liver fibrosis in T2DM patients by binary logistic regression

With the occurrence of NAFLD as the dependent variable, corrected for gender, BMI, GGT, CysC, HDL, HOMA-IR (CP), 25-(OH) D as the independent variables.

(Table 3), binary Logistic regression analysis showed that BMI(β = 0.136, *p* = 0.004), GGT(β = 0.037, *p* = 0.001), CysC(β = − 1.41, *p* = 0.002), HDL(β = − 1.347, *p* = 0.000),

**Table 3:** Influencing factors of NAFLD in T2DM patients

	β	OR	95%CI	p value
Sex	−0.315	0.73	0.416–1.281	0.272
BMI	0.136	1.146	1.045–1.257	0.004
GGT	0.037	1.038	1.016–1.061	0.001
CysC	−1.41	0.244	0.099–0.600	0.002
HDL	−1.347	0.26	0.097–0.698	0.007
HOMA-IR (CP)	0.261	1.298	1.046–1.612	0.018
25-(OH) D	−0.051	0.951	0.914–0.989	0.011

*BMI* Body mass index, *GGT* Glutamyltransferase, *CysC* Cystatin c, *HDL* High density lipoprotein, *Homa-IR (CP)* Homeostasis model assessment for insulin resistance(C-peptide), *25-(OH) D* 25- hydroxyvitamin D, *CI* Confidence interval, *OR* Odds ratio

HOMA-IR (CP)(β = 0.261,*p* = 0.018),25-(OH)D(β = − 0.051,*p* = 0.011) were the influencing factors of NAFLD in T2DM patients. With the occurrence of NFS as the dependent variable, gender was corrected, BMI, CysC, diabetic duration, 25-(OH) D as independent variables (Table 4), binary Logistic regression analysis showed that BMI(β = 0.129,*p* = 0.017),CysC(β = 3.564,*p* = 0.000),diabetic duration(β = 0.057,*p* = 0.046),25-(OH) D(β = − 0.072,*p* = 0.044) were the influencing factors of progressive liver fibrosis in T2DM with NAFLD patients.

**Table 4:** Influencing factors of progressive liver fibrosis

	β	OR	95%CI	p value
Sex	0.015	1.015	0.479–2.154	0.968
BMI	0.129	1.138	1.024–1.266	0.017
CysC	3.564	35.297	7.453–167.171	0.000
25-(OH) D	−0.072	0.931	0.868–0.998	0.044
Diabetic duration	0.057	1.059	1.001–1.120	0.046

*BMI* Body mass index, *CysC* Cystatin c, *25-(OH) D* 25- hydroxyvitamin D, *CI* Confidence interval, *OR* Odds ratio

### The prevalence of NAFLD and progressive liver fibrosis in low, medium and high levels of 25-(OH) D

**Fig. 1:**
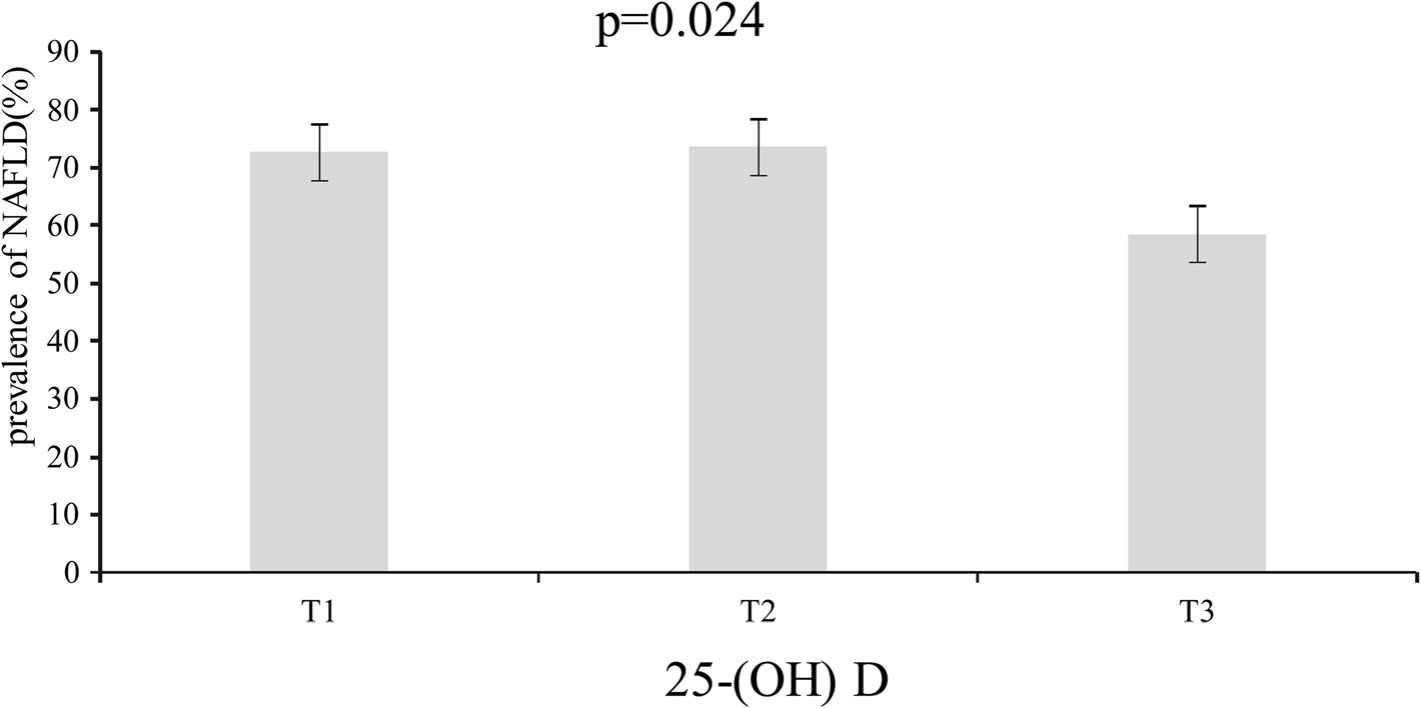
showed the relevance of the incidence of NAFLD with 25-(OH) D levels. 339 T2DM patients pooled together and analyzed according to the three-point numbers of 25-(OH) D levels. The results suggested that the incidence of NAFLD showed a significant decrease trend with levels of 25-(OH) D increasing(*p* < 0.05)

**Fig. 2:**
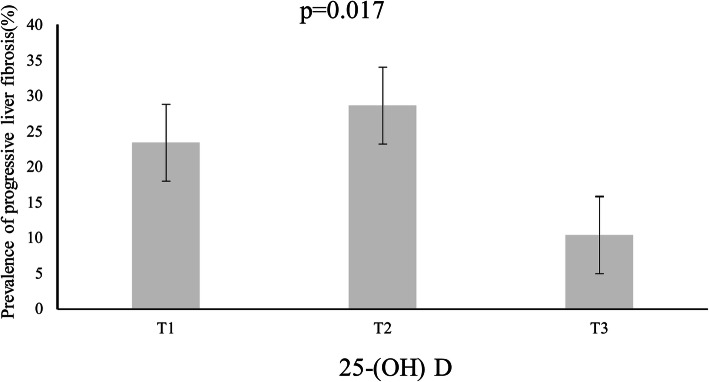
showed the relevance of the incidence of progressive liver fibrosis with 25-(OH) D levels. 231 T2DM with NAFLD patients pooled together and analyzed according to the three-point numbers of 25-(OH) D levels. The incidence of progressive hepatic fibrosis displayed a decrease trend under the increased levels of 25-(OH) D (*p* < 0.05). Annotation: NAFLD,Nonalcoholic fatty liver disease;25-(OH) D,25- hydroxyvitamin D;T1,T2,T3,three-point numbers

In 339 T2DM patients,they were divided into three groups according to triquantile levels of 25-(OH) D: T1 < 12.747, 12.747 ≤ T2 ≤ 17.777, T3 > 17.777 ng/mL,it was no difficult to find that prevalence of NAFLD presented a significant downward trend with levels of 25-(OH) D increasing (*P* < 0.05) (Fig. 1). In 231 T2DM complicated with NAFLD patients, they were divided into three groups according to the triquantile of 25-(OH) D levels, namely T1 < 12.427, 12.427 ≤ T2 ≤ 17.213,

T3 > 17.213 ng/mL.We could draw a conclusion that the differences among three groups were statistically significant (*P* < 0.05) (Fig. 2). At the high levels of 25-(OH) D, the prevalence of progressive liver fibrosis decreased significantly, while at the medium levels of 25-(OH) D, the prevalence of progressive liver fibrosis increased slightly.

## Discussion

NAFLD is considered as the manifestation of IR and metabolic syndrome in liver, which is a group of chronic inflammatory liver diseases that can develop into steatohepatitis and fibrosis, and an independent risk factor for T2DM and cardiovascular diseases [20, 21]. Liver biopsy is the “gold standard” in the process of diagnosing NAFLD and liver fibrosis, which is a invasive examination, so many scholars [8, 9] have proposed the use of NFS as evaluation of liver fibrosis, and defined NFS ≥ 0.676 as progressive liver fibrosis. Up to now, the theory of “second strike” is still the classic pathogenesis of NAFLD, which holds that the disorder of lipid metabolism leads to the accumulation of TG in liver cells, resulting in the formation of “first strike”, and then after the “second strike” of IR,inflammatory.

factors, oxidative stress, etc., liver inflammation and even fibrosis will occur. While vitamin D may improve IR by acting on vitamin D receptor through its active form 25-(OH) D, and reduce the release of pro-inflammatory factors and improve hepatocyte apoptosis and liver inflammation by inhibiting nuclear factor-κb and toll-like receptor pathway in liver. At the same time, activation of vitamin D receptor signaling pathway can inhibit the proliferation of hepatic stellate cells and improve liver fibrosis in NAFLD patients [22, 23]. At present, the research on active vitamin D and IR-related diseases has become a hot spot in endocrine field. Della Corte C et al. [24] found that vitamin D supplementation could improve IR of NAFLD patients.

More and more studies had shown that the low levels of vitamin D were closely related to the occurrence and development of NAFLD [13, 25, 26]. Lai et al. [27] found that compared with healthy control group, the levels of 25-(OH) D were significantly lower in NAFLD patients,especially in patients with moderate and severe NAFLD. Yang et al. [13] also found that serum 25-(OH) D decreased significantly in patients with liver fibrosis. After correcting for gender, age, BMI, FBG and other factors, 25-(OH) D was negatively correlated with NAFLD and progressive liver fibrosis.

Active vitamin D supplementation could alleviate progression of NAFLD [28].

The above-mentioned clinical research subjects were all non-diabetic health check-up people. Hosny etal [29] pointed out that the vitamin D levels were decreased in patients with T2DM with NAFLD. Vitamin D supplementation could produce beneficial effects [30]. This study found that patients in T2DM with NAFLD group were younger, shorter diabetes course,and the values of BMI,waist circumference, ALT, AST,GGT,FBG, TG,TC, HOMA-IR (CP) and HBA1C values were all higher in T2DM with NAFLD group,suggesting that T2DM with NAFLD patients were more prone to metabolic abnormalities and IR. The values of CR, CysC, HDL and 25-(OH) D in T2DM with NAFLD patients were lower than those in T2DM patients alone. BMI, GGT, CysC, HDL, HOMA-IR (CP) and 25-(OH) D were the influencing factors of NAFLD in T2DM patients. It could be seen that 25-(OH) D was an protective factor of NAFLD, which was basically consistent with the research results of Yang et al. [31].

Studies found that supplementation of Vitamin D can improve hepatic steatosis and insulin resistance by up-regulating vitamin D receptor and overexpression of hepatocyte nuclear factor 4 α [32]. At the same time, 1,25 -(OH) 2D3 could improve the liver function of mice with non-alcoholic fatty liver fibrosis induced by methionine-choline deficiency diet, and it may slow down the progress of liver fibrosis by regulating α-SMA and type I collagen genes [33]. In this study,it was no difficult to find that the patients in progressive liver fibrosis subgroup were older, and the values of BMI, AST, UA, HOMA-IR (CP) were higher,while values of HDL and 25-(OH) D were lower in progressive liver fibrosis subgroup than simple T2DM group and non-progressive liver fibrosis subgroup. Meanwhile,we also came to the conclusion that an obvious decrease trend of NAFLD and progressive liver fibrosis incidence was accompanied by the increasing of 25-(OH) D levels,which was consistent with the research results of Yu [34]. In addition, this study also discovered that apart from BMI in NFS formula, CysC, diabetes course were positively correlated with progressive liver fibrosis in T2DM with NAFLD patients, while 25-(OH) D was negatively correlated with progressive liver fibrosis. It could be seen that 25-(OH) D was an protective factor for progressive liver fibrosis. This was basically consistent with the findings of Yang et al. [31] that CysC and course of disease were risk factors of liver fibrosis, while ALT, ApoB and 25-(OH) D were protective factors of liver fibrosis.

However, Anty etal [14] considered that the low levels of 25-(OH) D had nothing to do with NAFLD,we speculated that the differences between studies may be related to the difference in NAFLD diagnosis method and detection method. Anty etal chose liver biopsy to diagnose NAFLD and used Elisa kit to detect 25-(OH)D,while We chose liver ultrasound examination to diagnose NAFLD and used high performance liquid chromatography-tandem mass spectrometry to detect 25-(OH)D.Furthermore,in this study, the one can be mentioned here is that the number of sample is limited. The influence of various chronic complications such as diabetes, cardiovascular and cerebrovascular diseases, hypoglycemic drugs and lipid-regulating drugs on vitamin D levels were not considered, and further work needed to be further analyzed in the future. Different methods in liver fibrosis methods might affect the inconsistency of conclusions. Therefore, a thorough understanding of the results in this study needed to consider clinical situation.

## Conclusions

To sum up, based on the above mentioned contents, it is no difficulty to find that the decrease of serum 25-(OH) D levels in T2DM with NAFLD patients were related to progressive liver fibrosis, and the low levels of 25-(OH) D were the risk factor of NAFLD and progressive liver fibrosis. In order to remind clinicians to pay attention to the people of 25-(OH) D deficiency or deficiency in T2DM with NAFLD patients, and be alert to the risk of developing progressive liver fibrosis in these patients.

## Data Availability

All data generated or analyzed during this study are included in the article. The data that support this study are available from the corresponding author only upon reasonable request, once the study has been published.

## Abbreviations

NAFLD: Nonalcoholic fatty liver disease
T2DM: Type 2 diabetes mellitus
NFS: Nonalcoholic fatty liver disease fibrosis score
25-(OH)D: 25-hydroxyvitamin D
IR: Insulin resistance
NAS: Nonalcoholic steatosis
NASH: Non-alcoholic steatohepatitis
SBP: Systolic pressure
DBP: Diastolic pressure
FCP: Fasting C-peptide
Homa-IR (CP): Modified homeostasis model assessment for insulin resistance(C-peptide)
AST: Asparticacid aminotransferase
ALT: Alanine aminotransferase
GGT: Glutamyltransferase
CR: Creatinine
UA: Uric acid
CysC: Cystatin C
FBG: Fasting blood glucose
TG: Triglyceride
TC: Total cholesterol
HDL: High density lipoprotein
LDL: Low density lipoprotein
ALB: Albumin
HbA1c: Glycosylated hemoglobin
PLT: Platelet
SD: Standard deviation
OR: Odds ratio
CI: Confidence interval
